# Dynamics of a Mosquito Egg-Larvae Model with Seasonality

**DOI:** 10.1007/s11538-023-01238-0

**Published:** 2023-12-18

**Authors:** Jesús Bellver-Arnau, Alessandro Margheri, Carlota Rebelo

**Affiliations:** 1 Laboratoire J.-L. Lions, CNRS, Inria, Université de Paris, Sorbonne Université, 75005 Paris, France; 2 Present Address: Centre d’Estudis Avançats de Blanes (CEAB), Consejo Superior de Investigaciones Científicas (CSIC), Carrer d’Accés a la cala Sant Francesc 14, 17300 Blanes, Spain; 3 Departamento de Matemática and CMAFcIO, Faculdade de Ciências, Universidade de Lisboa, 1749-016 Campo Grande, Lisbon, Portugal; 4 Departamento de Matemática and CEMAT-Ciências, Faculdade de Ciências, Universidade de Lisboa, 1749-016 Campo Grande, Lisbon, Portugal

**Keywords:** Mosquito life cycle, Systems with seasonality, Periodic attractor, Monotone systems, Vectorial reproduction number, 34C25, 34D20, 92D25

## Abstract

We propose a two stages mosquito egg-larvae model with seasonality as a simplification of a four stages one. For the simplified model we characterize the dynamics in terms of the vectorial reproduction number, $R_0$, obtaining extinction if $R_0\le 1$ and convergence to a unique positive periodic orbit if $R_0>1$. We illustrate each case with an example, by providing general conditions on the periodic coefficients for its occurrence. These examples are further developed using numerical simulations where the periodic parameters satisfy the conditions obtained. In the $R_0>1$ case, real climatic data is used for inferring the parameter behaviour. For the four stage system, using alternative oviposition rate functions, we present a result which generalizes others given for models with delays and even with diffusion to the case in which competition between the larvae is introduced. The analytical study of our initial four stages system when $R_0\ge 1$ remains open, since we were not able to prove that in this case the system is dissipative.

## Introduction

Mosquitoes are responsible for transmitting numerous diseases. Different genera transmit different pathogens. *Anopheles* mosquitoes transmit malaria, *Aedes* mosquitoes transmit several virus of the family *Flaviviridae*, such as dengue, Zika, yellow fever or West Nile fever, and *Culex* transmit the Japanese encephalitis virus and West Nile fever (Tolle 2009), to name a few. These diseases have a combined death toll of approximately 700,000 people annually (OrganizationWHO 2022), making the mosquito the most dangerous animal in the world, and a cause of concern for public health. Due to this, different vector control strategies have been used in the past while others are currently being developed: from the use of pesticides and the physical removal of breeding sites to more sophisticated techniques involving the release of modified vectors, such as the sterile insect technique (Dame et al. 2009; Lees et al. 2015; Bellini et al. 2013), or the use of the endosymbiotic bacterium *Wolbachia* (Mousson et al. 2012; O’Neill et al. 2018; Ong 2021; Tantowijoyo et al. 2020). In order to deploy a successful control strategy of any kind it is crucial to properly understand how seasonality affects the mosquito life cycle and its dependence on climatic conditions (Cailly et al. 2012; Gloria-Soria et al. 2022).

Although with minor variations, every mosquito genera goes through the same basic life cycle (Crans 2004). This life cycle is composed of two clearly distinct phases: an aquatic phase, as juveniles, and an aerial phase, as adults. The aquatic phase is on its turn composed of three stages: egg, larval and pupal. Due to the complexity of the topic and the numerous factors involved, many details about this life cycle remain unknown. Among the factors influencing in one way or another the development of the mosquito we find environmental ones, such as temperature, rainfall, humidity, abundance or lack of nutrients or the photoperiod (daylight length). On the other hand there are also intrinsic factors to the species such as larval competition (Reiskind and Lounibos 2009; Bara et al. 2015; Alto et al. 2005), hibernation mechanisms such as the diapause (a dormant state eggs of some mosquito species can enter to face adverse conditions Denlinger and Armbruster 2014) or the complex hatching rate response to the presence of larvae in the environment, where both stimulant and inhibitory mechanisms of the hatching coexist (Edgerly and Marvier 1992; Livdhal and Futterweit 1983).

Nevertheless, most of these factors, as complex as they may be, have something in common: they vary periodically during the year, and so does mosquito population. There are plenty of works in the literature correlating variations in temperature, usually temperature and rainfall, with bursts in mosquito population (Honório et al. 2009; Lana et al. 2018; Ewing et al. 2019; Tran et al. 2013), which in turn, can give rise to vector-borne disease epidemic outbreaks (Shen et al. 2015; Mukhtar et al. 2019; Pliego et al. 2017). On top, meta analysis shows that the main factor affecting the development rate of mosquitoes is temperature, and that other factors should never be considered to the exclusion of temperature (Couret and Benedict 2014). Temperature (or alternatively, rainfall in the regions closest to the equator), present a clear periodic variation throughout the year. Therefore, understanding, even in a simplified setting, the behaviour of mosquito population in a system with periodic coefficients can give some insight of the intricate puzzle of mosquito life cycle. The approach usually considered for mechanistic models of ODEs is to get some insights by numerical simulations of the models, calibrated for specific set of parameters (see e.g. Erickson et al. 2010, Wang et al. 2016, Cailly et al. 2012). Our aim here is rather to obtain rigorous general qualitative results for the dynamics of such systems, in the spirit of the ones presented in Abdelrazec and Gumel (2017). Accordingly, the main focus of this paper will be the study of a seasonal two stages mosquito egg-larvae model (in the following represented mathematically by variables (*E*) and (*L*) respectively), proposed as a simplification of a four stage model which appears to be less amenable to mathematical analysis, and whose dynamics we were not able to characterize. More precisely, we will address the following system

1 $$\begin{aligned} {\left\{ \begin{array}{ll} E'=b_E(t)L-d_E(t)E-h(t)E,\\ L'=h(t)E-d_L(t)L-c(t)L^2, \end{array}\right. } \end{aligned}$$

with fairly general and biologically meaningful assumptions on the *T*-periodic coefficients, assumed continuous, nonnegative with positive average on [0, *T*]. Therefore, no assumption on the causes or effects of the complex factors affecting the coefficients is made a priori. System (1) is the *T*-periodic version of the, analogous, but autonomous one studied in Strugarek et al. (2019), where the hatching rate depends on the larvae. That model was derived from the following four stage one, where also pupae (P) and adults (A) are considered:

2 $$\begin{aligned} {\left\{ \begin{array}{ll} E'=\beta _E A-d_E E- h E ,\\ L'=h E-d_L L-c L^2-\tau _L L ,\\ P'= \tau _L L-d_P P-\tau _P P ,\\ A'= \tau _P P-d_A A, \end{array}\right. } \end{aligned}$$

here the functions $d_i$, $i=E,L,P,A$, stand for the death rate at each life stage, $\tau _i$, $i=L,P$, for the transition rate from each life stage to the next, $\beta _E$ is the oviposition rate of adult mosquitoes, *h* the hatching rate which in that paper depended on *L* and *c* the strength of the intraspecific competition between the larvae. In system (1), the parameters that are denoted the same are equivalent to its counterparts in (2), whereas $b_E$ can be seen as an analogous to the oviposition rate of adult mosquitoes, $\beta _E$, for this simplified setting. For the sake of convenience, we will also call "oviposition rate" to $b_E$.

We point that, although the main focus will be on system (1), in our paper we also improve one of the results presented in Abdelrazec and Gumel (2017) for a four stage model, allowing intraspecific competition among larvae.

Our paper is organized as follows: in Sect. 2 we analyze the dynamics of system (1) and prove that if the vectorial reproduction number $R_0$ is less or equal than one, then eggs and larvae go extinct. Otherwise, there exists a unique periodic orbit which attracts all the positive solutions of the system. As we do not have an explicit expression for $R_0$ depending on the coefficients of the system, we determine conditions which guarantee that $R_0>1$ and after we give an example corresponding to $R_0\le 1$.

Section 3 presents some numerical simulations illustrating the aforementioned examples. For the $R_0>1$ case, two scenarios are presented, corresponding to a tropical and a temperate climate respectively. Climatic data (temperature and rainfall), thermal responses of *Ae. Albopictus*’ biological traits, obtained from Mordecai et al. (2017), Jia et al. (2016), and the rainfall-dependent modeling of the carrying capacity done in Erguler et al. (2016) are incorporated to obtain the periodic parameters used in the simulations.

In Sect. 4 we address briefly four stage seasonal models of mosquito population. For the model considered in Abdelrazec and Gumel (2017), which is a *T*- periodic variation of system (2) but with an oviposition rate term that saturates, we show that the characterization of the dynamics in terms of $R_0$ (extinction if $R_0\le 1$, convergence to a positive *T*-periodic orbit if $R_0>1$) holds also in the case of competition between larvae. This result generalizes the analogous one contained in the mentioned paper.

Unfortunately, we were not able to obtain a complete result for our original four stage model, since we could not prove that the system is dissipative when $R_0\ge 1.$ In particular the existence of a globally attracting *T*-periodic orbit for $R_0>1,$ although some numerical simulation suggest a positive answer, remains an open problem.

## Dynamics of System (1)

In this Section we are interested in the dynamics of solutions of system (1)

$$\begin{aligned} {\left\{ \begin{array}{ll} E'=b_E(t)L-d_E(t)E-h(t)E,\\ L'=h(t)E-d_L(t)L-c(t)L^2, \end{array}\right. } \end{aligned}$$

$E\ge 0$, $L\ge 0$ (standing for the amount of Eggs and Larvae respectively), where $b_E,\,d_E, \,d_L,\,h$ and *c* are continuous and *T*-periodic functions, $b_E,\,d_E, \,d_L$ and *h* are nonnegative, but not identically zero, and *c* is strictly positive. In Strugarek et al. (2019) an analogous autonomous model for mosquitoes’ eggs and larvae was considered but in which hatching depended in a nonlinear way on larval density. Here we opted to assume that hatching does not depend on larval density, but rather that all coefficients can be seasonally dependent as there is evidence on the impact of seasonality on the different coefficients of the system. In particular, we will focus on the dependencies of the parameters on temperature and precipitation, that we take from Mordecai et al. (2017), Jia et al. (2016), Erguler et al. (2016). Under these conditions, existence and uniqueness of solutions of associated Cauchy problems is guaranteed. Note that (0, 0) is an equilibrium of the system and that we have the following result, where the inequalities on vectors must be interpreted as holding component-wise:

### Lemma 2.1

Let (*E*(*t*), *L*(*t*)) be a solution of system (1) with initial condition $(E(0),L(0))\ge 0$. Let $I:=[0,\omega )$ be its maximal right domain of existence. Then $(E(t),L(t)) \ge 0$ for all $t\in I$.

### Proof

The statement follows immediately from the sign condition of the vector field on the boundary of the first quadrant, namely if $(E,L)\ge (0,0)$ then $E=0 \Rightarrow E'\ge 0$ and $L=0\Rightarrow L'\ge 0.$ $\square$

In the following, and for conciseness, we denote with a superscript *m* (respectively *M*) the minimum (respectively the maximum) of the functions in [0, *T*].

Now we prove that our system is dissipative.

### Lemma 2.2

The solutions of (1) are defined for each $t\ge 0$. Moreover, there exists a $\mathcal{K}>0$ such that $E(t)+L(t)\le \mathcal{K}$ for each *t* sufficiently large.

### Proof

We have

$$\begin{aligned} \frac{d}{dt}(E+L)&=b_{E}(t)L-d_{E}(t)E-d_{L}(t)L-c(t)L^{2}\\&=-d_{E}(t)(E+L)+(b_{E}(t)+d_{E}(t)-d_{L}(t))L-c(t)L^{2}\\&\le -d_{E}(t)(E+L)+ U_{M}, \end{aligned}$$

where $$ \ U_{M} $$ is the maximum of $$ (b_{E}^{M}+d_{E}^{M}-d_{L}^{m})L-c^{m}L^{2}$$ for $$L\ge 0$$.

Now, using Halanay (1966, Theorem 3.1) and recalling that $d_E$ is not identically zero, we can conclude that all the solutions of

$$\begin{aligned} y'=-d_{E}(t)y+ U_{M} \end{aligned}$$

converge to a periodic one $\bar{y}$. Hence, there exists $\epsilon >0$ such that given a solution *y*(*t*) of this equation, there exists $\bar{t}$ such that for each $t\ge \bar{t}$ we have $y(t)\le \max \bar{y}+\epsilon := \mathcal{K}$. By comparison, considering the solution with $y(0)=E(0)+L(0),$ for each $t\ge \bar{t}$ we have $(E+L)(t)\le \mathcal{K}$. Finally, taking into account the previous lemma all the solutions are nonnegative and hence all the solutions are bounded in the future and consequently defined in the future, that is, for each $t\ge 0$. $\square$

From lemmas 2.1 and 2.2 we get the following:

### Corollary 2.3

Let (*E*(*t*), *L*(*t*)) be a solution of system (1) with initial condition $(E(0),L(0))\ge 0,\,$ $(E(0),L(0))\ne 0.$ Then $(E(T),L(T))>0$.

### Proof

Since the origin $(E,L)=(0,0)$ is an equilibrium of system (1), by uniqueness of solution of the initial value problem for such system, under the assumption of the corollary we have $(E(t), L(t))\ne (0,0)$ for all $t\in [0,+\infty ).$ If $E(0)>0$ and $L(0)>0$, taking into account the following inequalities

3 $$\begin{aligned} {\left\{ \begin{array}{ll} E'\ge -d_E(t)E-h(t)E\\ L'\ge -d_L(t)L-c(t)L^2 \end{array}\right. } \end{aligned}$$

we get that $E(t)>0$ and $L(t)>0$ for any $t\ge 0$, and the thesis follows. If $E(0)>0,\,L(0)= 0,\,$ let

$$\begin{aligned} t_L=\sup \{ t\in [0, T]:\,h(\tau )=0,\,\, \text{ for } \text{ all }\, \tau \in [0, t]\}.\, \end{aligned}$$

Since *h* has positive average on [0, *T*],  it is $t_L<T.$ We note that $L(t)=0$ on $[0, t_L]$ and claim that there exists $\hat{t}\in (t_L,T)$ such that $L(\hat{t})>0.$ Otherwise, by contradiction, it should be $0=L'(t)=h(t)E(t)$ on $(t_L,T),$ which is absurd, since there exists a positive $\delta$ such that $t_L+\delta <T$ and $h(t)E(t)>0$ for $t\in (t_L, t_L+\delta ).$ Then, by comparison, using the second inequality in (3) and the initial condition $L(\hat{t})>0,$ we get that $L(T)>0$, and the proof for this case is concluded. The case $L(0)>0,\, E(0)= 0$ is dealt with analogously. $\square$

From the previous Corollary we conclude that the *T*-Poincaré map $\Psi _T:[0,+\infty )^2\rightarrow [0,+\infty )^2$ of system (1) given by

$$\begin{aligned} (E_0,L_0)\rightarrow \Psi _T (E_0,L_0):= (E(T;E_0,L_0),L(T;E_0,L_0)), \end{aligned}$$

where we denote by $(E(\cdot ;E_0,L_0),L(\cdot ;E_0,L_0))$ the solution of system (1) such that $(E(0;E_0,L_0),L(0;E_0,L_0))=(E_0,L_0),$ is well defined and is a positive map. Moreover, we recall that, since the vector field associated to system (1) is smooth in the (*E*, *L*) variables, the same property holds for the map $\Psi _T.$

We give now conditions for the extinction of *E* and *L* or for the existence of a globally asymptotically stable periodic solution of the system (1). As in Abdelrazec and Gumel (2017), we consider the notion of basic reproduction number for periodic systems $R_0$ (see Bacaër and Guernaoui 2006, Wang and Zhao 2008), which in this case can be referred as the vectorial reproduction number. This reproduction number is the spectral radius of the next generation operator of the population.

With this purpose, we consider the linearization of (1) around the origin (0, 0)

$$\begin{aligned} {\left\{ \begin{array}{ll} u'=b_E(t)v-(d_E(t)+h(t))u\\ v'=h(t)u-d_L(t)v \end{array}\right. } \end{aligned}$$

and set (see Wang and Zhao 2008)

$$\begin{aligned} F(t)&=\left[ \begin{array}{cc} 0 &{} b_E(t)\\ 0 &{} 0 \end{array} \right] \hspace{1cm} V=(t)\left[ \begin{array}{cc} d_E(t)+h(t) &{} 0\\ -h(t) &{} d_L(t) \end{array} \right] . \end{aligned}$$

By (Wang and Zhao 2008, Theorem 2.2), the spectral radius $\rho (\Phi _{F-V})$ of the monodromy matrix associated with the previous linear system satisfies

$$\begin{aligned} \rho (\Phi _{F-V})>1 \text{ iff } R_0>1,\,\, \rho (\Phi _{F-V})=1 \text{ iff } R_0=1 \text{ and } \rho (\Phi _{F-V})<1 \text{ iff } R_0<1. \end{aligned}$$

Recall that the monodromy matrix of a *T*- periodic linear system is its fundamental matrix evaluated at $t=T.$

### Theorem 2.4

We have that

- If $$R_0\le 1$$ then $$(E(t),L(t))\rightarrow (0,0)$$.
- If $R_0>1$ then there exists exactly a unique periodic solution $(E^*(t),L^*(t))$which is globally asymptotically stable in $\textbf{R}_+^2\setminus \{(0,0)\}$.

### Proof

The result follows from Zhao (2017, Theorem 3.1.2) (see also Zhao 2017, Theorem 2.3.4). In fact if we consider the vector field

$$\begin{aligned} G(t,E,L)=(G_1(t,E,L), G_2(t,E,L))\\ :=(b_E(t)L-(d_E(t)+h(t))E, h(t)E-d_L(t)L-c(t)L^2) \end{aligned}$$

we have that

(i) $G_1(t,0,L)\ge 0$ if $L\ge 0$ and $G_2(t,E,0)\ge 0$ for $E\ge 0$ for each $t\ge 0$,
(ii) $$\frac{\partial G_1}{\partial L}\ge 0$$ and $$\frac{\partial G_2}{\partial E}\ge 0$$,
(iii) *G*(*t*, *E*, *L*) is strictly subhomogeneous, that is
  $$G(t, \alpha E, \alpha L)\ge \alpha G(t,E,L)\,\, \text{ and }\,\, G(t, \alpha E, \alpha L)\ne \alpha G(t,E,L)$$
  for $$\alpha \in (0,1)$$ and $$t\ge 0,\,\,E>0,\, \,L>0.$$

We mention that as stated in Zhao (2017, Page 67), (iii) implies that the Poincaré map is strictly subhomogeneous. Also by Corollary 2.3 we know that the Poincaré operator $\Psi _T(E,L)$ is positive. In order to prove that $\Psi _T$ is also strongly monotone, we start by observing that the Jacobian matrix of this operator, $D\Psi _T(E,L),$ in a point $(E_0,L_0)$ is the value at time $t=T$ of the fundamental matrix *X*(*t*) of the linearization of system (1) around $(E(t;E_0,L_0), L(t;E_0,L_0)),$ namely, *X*(*t*) satisfies

$$\begin{aligned} X'(t)= \left[ \begin{array}{cc} -(d_E(t)+h(t)) &{} b_E(t)\\ h(t) &{} -(d_L(t)+2c(t)L(t;E_0,L_0)) \end{array} \right] X(t),\quad X(0)=I_2, \end{aligned}$$

where $I_2$ denotes the identity matrix of order two.

Reasoning as in the proof of Corollary 2.3, but considering now the initial conditions (1, 0) and (0, 1),  the columns of $I_2$, we obtain that all the elements of $D\Psi _T(E_0,L_0),$ are positive, for any $(E_0,L_0)\ge 0$ with $(E_0,L_0)\ne (0,0)$. Taking into account this fact we get that if $(E_1,L_1)\gneq (E_2,L_2)\ge (0,0), \,\,(E_i,L_i)\ne (0,0),\,\,i=1,2,\,$ then $\Psi _T(E_1,L_1)>\Psi _T(E_2,L_2)$, since

$$\begin{aligned}{} & {} \Psi _T(E_1,L_1)-\Psi _T(E_2,L_2)\\{} & {} \quad =\left( \int _0^1 D\Psi _T(sE_1+(1-s)L_1, sE_2+(1-s)L_2) \,ds\right) (E_1-E_2,L_1-L_2)>0, \end{aligned}$$

where the vector $(E_1-E_2,L_1-L_2)$ must be intended as a row vector. Thus, to get the strong monotonicity condition of the Poincaré operator $\Psi _T$ we do not need the irreducibility condition on the Jacobian of the vector field $(E,L)\rightarrow G(t,E,L)$, an assumption that in our setting, in general, does not hold since we allow some of the time dependent coefficients to be zero (but not identically equal to zero). We conclude that we can apply Zhao (2017, Theorem 3.1.2) and the result follows. $\square$

The previous result determines the dynamics of system (1) as a function of $R_0$, but in general it is not possible to give an explicit expression for this vectorial reproduction number, which highlights its dependence on the periodic coefficients. Hence, in Sects. 2.1 and 2.2, we present two general examples which provide some conditions on the periodic coefficients which, according to Theorem 2.4, are sufficient to get respectively, $R_0>1$ and $R_0\le 1$. Indeed, in the first example we prove the existence of a unique globally asymptotically stable *T*-periodic orbit, in the second we show that extinction occurs. We point out that, in the case $R_0>1$, these conditions are a generalization of the ones considered for the autonomous case in Strugarek et al. (2019).

### Example 1: $R_0>1$

In what follows, we will assume the following conditions on the coefficients

4 $$\begin{aligned} b_E(t_*)&=0 \text{ for } \text{ some } t_* \Longrightarrow d_E(t_*)=0 \text{ and } d_L(t_*)=0 \end{aligned}$$

5 $$\begin{aligned} h(t_*)&=0 \text{ for } \text{ some } t_* \Longrightarrow b_E(t_*)=0 \end{aligned}$$

6 $$\begin{aligned}&\sup _{t\in \mathcal{T}}\dfrac{d_{L}}{b_{E}} < \inf _{t\in \mathcal{T}} \dfrac{h}{d_{E}+h} \text{ where } \mathcal{T}=\left\{ t: b_E(t)\ne 0 \text{ and } h(t)\ne 0\right\} . \end{aligned}$$

It is important to note that if we assume that all the coefficients are positive periodic functions, we only remain with condition (6) which is analogous to condition (4) in Strugarek et al. (2019) for the autonomous model.

Now it is useful to extend the system to all the plane. We consider

7 $$\begin{aligned} {\left\{ \begin{array}{ll} E'&{}= b_{E}(t)L-d_{E}(t)E-h(t)E \\ L'&{}= h(t)E-d_{L}(t)L- c(t,L), \end{array}\right. } \end{aligned}$$

where $c(t,L):=$ $$\left\{ \begin{array}{ll} c(t)L^{2}, \ \ L \ge 0 \\ -c(t)L^{2}, \ \ L<0 \end{array} \right. $$. This extension is a mathematical construction that, although meaningless from a biological point of view, will allow us to prove Proposition 2.5, which does have a biological meaning.

We notice that the extended vector field, which we still denote by *G*(*t*, *E*, *L*),  is $C^1$ (but not $C^2$) in the whole (*E*, *L*) plane, and we indicate with *DG*(*t*, *E*, *L*) its Jacobian matrix in the (*E*, *L*) variables.

In what follows we denote by $Q_i,\,\,i=1, 2,3,4$ the closed quadrants in the (*E*, *L*) plane, counted in a counterclockwise sense starting from $Q_1:=[0, +\infty )^2$, and we denote by $Q_i^\circ ,\,\,i=1,2,3,4,\,$ their interior.

Repeating the proof of Lemma 2.2 for initial conditions in $Q_2$ we conclude that those solutions are defined in the future. Moreover, with this extension we have that if (*E*(*t*), *L*(*t*)) is a solution of (7) in $Q_3$, $(-E(t),-L(t))$ will be a solution of the same system in $Q_1$ and vice-versa. Analogously for solutions in $Q_2$ and $Q_4$. As a consequence, the *T*- Poincaré operator associated to system (7), $$\Psi _T: \mathbb {R}^2 \rightarrow \mathbb {R}^2 \ $$ is well defined, and, since it is an extension to the plane of the *T*-Poincaré operator of system (1) we still denote it by $\Psi _T.$ Due to the aforementioned regularity of the vector field *G*,  the map $\Psi _T$ is $C^1(\mathbb {R}^2)$.

Let us now analyze the dynamics of system (7) around the equilibrium (0, 0).

#### Proposition 2.5

Consider system (7) and assume (4), (5) and (6). Then, the origin is a saddle point. Moreover, the origin is a repellor with respect to the set $Q_1{\setminus }\{(0,0)\}$, and hence $R_0>1$.

#### Proof

We will apply Coelho et al. (2021, Lemma 3.3). We have,

$$\begin{aligned} DG(t,0,0)= \left[ \begin{matrix} -d_{E}(t)-h(t) &{} b_{E}(t) \\ h(t) &{} -d_{L}(t)\\ \end{matrix} \right] , \end{aligned}$$

where $b_{E}\ge 0$ and $h\ge 0$ but not identically zero. Also

$$\begin{aligned} \int ^{T}_{0} \left( -d_{E}(t)-h(t) -d_{L}(t) \right) dt =- \int ^{T}_{0}\left( d_{E}(t)+h(t)+d_{L}(t) \right) dt < 0 . \end{aligned}$$

Let us prove that there are $\alpha _1$, $\alpha _2 \in \mathbb {R}^+$ and $t^{*} \in \left[ 0,T \right]$ as in Coelho et al. (2021, Lemma 3.3). We consider

$$\begin{aligned} B_1(t)&=\alpha _1 (-d_{E}(t)-h(t))+ \alpha _2 h(t) \\ B_{2}(t)&= -\alpha _2d_L(t)+\alpha _1b_E(t) \end{aligned}$$

and choose $\alpha _1$ and $\alpha _2$ adequately. From (4) and (5), if for some $\bar{t}$ we have $b_E( \bar{t} )=0$ then both $d_L(\bar{t})$ and $d_E(\bar{t})$ are zero and if $h(\bar{t})=0$ then $b_E(\bar{t})=0$. We conclude that if $h(\bar{t})=0$ then for $i=1,2$, $B_i(\bar{t})=0$. In the case $b_E(\bar{t})=0$ we have that $B_2(\bar{t})=0$ and $B_1(\bar{t})\ge 0$ if $\alpha _2\ge \alpha _1$. Let us now consider the cases in which both $b_E(t)\ne 0$ and $h(t)\ne 0$, that is when $t\in \mathcal{T}$. As (6) is satisfied we have that there exist $\alpha _1$ and $\alpha _2$ with $\alpha _2>\alpha _1$ such that

$$\begin{aligned} \dfrac{d_{L}(t)}{b_{E}(t)}< \dfrac{\alpha _1}{\alpha _2} < \dfrac{h(t)}{d_{E}(t)+h(t)}. \end{aligned}$$

for all $t\in \mathcal{T}$. Applying the aforementioned result in Coelho et al. (2021, Lemma 3.3), we conclude that the origin is a saddle point for system (7). In particular, by the stable manifold theorem for fixed points (see Guckenheimer and Holmes 1983, Theorem 1.4.2), $\Psi _T$ has a one-dimensional stable manifold and a one-dimensional unstable manifold. These, are tangent at (0, 0), respectively, to the one-dimensional stable subspace and to the one-dimensional unstable subspace of $\Phi _T:=D\Psi _T(0,0)$, where $\Phi _T$ is the Poincaré operator of the linearization of system (7) in (0, 0).

To prove that the origin is a repellor of system (7) in $Q_1\setminus \{(0,0)\}$ it is now sufficient to observe that a half-line of the stable manifold of $\Phi _T$ is contained in $Q^{\circ }_4$, whereas a half-line of the unstable one is contained $Q^{\circ }_1$. These claims follow directly from the Perron-Frobenius theorem. In fact, by comparison between the linearized system and system (7) we get $\Phi _T(1,0)\ge \Psi _T(1,0)$ and $\Phi _T(0,1)\ge \Psi _T(0,1)$, and by Corollary 2.3 we get $\Psi _T(1,0)>0$ and $\Psi _T(0,1)>0$. We conclude that the linear map $\Phi _T$ is positive, and the Perron-Frobenius theorem applies so that the eigenvectors associated to the dominant eigenvalue are in the first quadrant and the others are not. $\square$

### Example 2: $R_0\le 1$

Next, we provide an example showing that if $d_L$ is positive when *h* is zero, in which case either (4) or (5) do not hold, there may be extinction for system (1). Here, our aim is not focused on the choice of a realistic set of periodic parameters, but rather on showing the qualitative behaviour of the system in a case where $R_0\le 1$. Nevertheless, in a realistic setting, it is reasonable to expect $d_L>0$ when both $h=0$ and $b_E=0$, contrary to what is assumed in condition (4) and (5) of Example 1. This should be so, for instance, in case of a diapausing mosquito species during winter. More general conditions guaranteeing extinction as well as their biological consistency for the egg-larvae model will not be investigated here (but see Remark 2.6 below).

Let $\bar{b}_E$, $\bar{d}_E$, $\bar{d}_L$ and $\bar{h}$ be fixed positive constants such that

8 $$\begin{aligned} \frac{\bar{d}_L}{\bar{h}}<\frac{\bar{b}_E}{\bar{d}_E+\bar{h}}. \end{aligned}$$

We will choose $t_i\in (0,T), \,i=1,\ldots , 5$, with $t_i<t_{i+1}.$ We construct the continuous coefficients of the system as follows: $b_E(t)=\bar{b}_E,\,d_E(t)= \bar{d}_E,\, h(t)=\bar{h}$ on $[0, t_1]\cup [t_5, T],$ $b_E(t)=d_E(t)= h(t)=0$ on $[t_2, t_4]$ and $b_E,\,d_E,\,h$ are linear on $[t_1, t_2]\cup [t_4, t_5]$. Finally $d_L(t)=\bar{d}_L$ on $[0, t_2]\cup [t_5, T],$ $d_L(t)=0$ on $[t_3, t_4]$ and $d_L$ is linear on $[t_2, t_3]\cup [t_4, t_5].$ So, on $(t_2,t_3)$ these coefficients are zero except $d_L$. By construction and if $E> \frac{\bar{b}_E}{\bar{d}_E+\bar{h}} L$ on $[0,t_2)\cup (t_4, T]$ it is $\dot{E}\ge 0$ iff $E\le \frac{\bar{b}_E}{\bar{d}_E+\bar{h}}$ and on $[0,t_1)\cup (t_4, T]$ it is $\dot{L}>0$ iff $E\ge \frac{\bar{d}_L}{\bar{h}}.$ We will show that it is possible to choose suitably the times $t_i$ and the period *T*, in such a way that the linear system corresponding to these coefficients, that is, system (1) with $c(t)=0$ for all $t\in [0,T]$, has positive solutions which go to zero. By comparison, (1) will have too.

Let us consider

9 $$\begin{aligned} {\left\{ \begin{array}{ll} E'=b_E(t)L-d_E(t)E-h(t)E,\\ L'=h(t)E-d_L(t)L, \end{array}\right. } \end{aligned}$$

and choose

$$P_0=(E_0,L_0)\in \Gamma _1:= \left\{ (E,L)\in Q_1^\circ \,:\, E> \frac{\bar{b}_E}{\bar{d}_E+\bar{h}} L\right\} .$$

Then, we can fix $t_1$ in such a way that $\Phi _t(P_0)\in \Gamma _1$ for any $t\in [0, t_1]$. It follows that *E*(*t*) is strictly decreasing and *L*(*t*) is strictly increasing $t\in [0, t_1].$ Next, we choose $t_2$ in such a way that $E(t_2)<E_0$ (it is not necessary here that $\Phi _{t_2}(P_0)\in \Gamma _1$).

On $[t_2, t_3]$ system (9) becomes $E'=0,\,L'=-d_L(t) L,$ and we can fix $t_3$ in such a way that

$$\Phi _{t_3}(P_0)\in \Gamma _2:= \left\{ (E, L)\in \Gamma _1\,:\, E>\frac{E_0}{L_0}L\right\} ,$$

that is $\Phi _{t_3}(P_0)$ lies in the interior of the first quadrant, below the ray $\{\lambda P_0:\, \lambda >0\}$. Of course, we have $E(t_3)=E(t_2)<E_0$. On $[t_3, t_4]$ all solutions are constant. On $[t_4, T]$   *E*(*t*) is strictly decreasing and *L*(*t*) is strictly increasing and we can choose $t_5$ in such a way that $\Phi _{t_5}(P_0)\in \Gamma _2$. Finally, since on $[t_5, T]$ there are no positive equilibria of the corresponding linear autonomous system, we can choose *T* in such a way that $\Phi _T(P_0)=\lambda \Phi _0(P_0)=\lambda P_0,$ with $0<\lambda <1$. But then $Q_1^\circ \ni \Phi ^k_T(P_0)=\lambda ^k P_0\rightarrow (0,0)$ as $k\rightarrow +\infty$, and the same will hold for $Q_1^\circ \ni \Psi _T^k(P_0)\le \Phi ^k_T(P_0),$ (the inequality is to be understood component-wise). Taking into account the Perron-Frobenius theorem (Smith and Waltman 1995, Theorem A.4), we have that $\lambda$ is the dominant eigenvalue of the positive, nonsingular and orientation preserving operator $\Phi _T.$ We conclude that both Floquet multipliers of (0, 0) (thought of as a *T*-periodic orbit) are in (0, 1). By comparison with the linearized system, we have that the origin is globally stable in the first quadrant for system (1).

#### Remark 2.6

The previous construction can be extended to a more general framework. One can assume that the coefficients $b_E(t), \,d_E(t),\,d_L(t),\,h(t)$ are positive on $[0,t_1]\cup [t_5, T]$, not necessarily constant, replacing (8) with the following:

$$\begin{aligned} \max _{[0,t_1]\cup [t_5, T]} \frac{{d}_L(t)}{ h(t)}<\min _{[0,t_1]\cup [t_5, T]} \frac{{b}_E(t)}{{d}_E(t)+ h(t)}, \end{aligned}$$

and defining $\Gamma _1$ as

$$\begin{aligned} \Gamma _1:= \left\{ (E,L)\in Q_1^\circ :\, E> \max _{[0,t_1]\cup [t_5, T]} \frac{{b}_E(t)}{{d}_E(t)+ h(t)}L\right\} . \end{aligned}$$

Also, the linearity of the coefficients in the intervals on which they decrease to zero or increase from zero can be dropped. Finally, instead of choosing suitably the $t_i$ given the coefficients, one could fix the times $t_i$ and the period *T* and investigate if there are suitable choices of the coefficients which allow to carry out our construction. We will not pursue this topic here.

#### Remark 2.7

If we allow $d_L$ to be strictly positive on [0, *T*],  and assume that $b_{E}^{M}+d_{E}^{M}-d_{L}^{m}<0,$ then, by the proof of Lemma 2.2, we see that the origin is globally stable in $Q_1$ for system (1). Hence, we get another instance in which $R_0\le 1.$ However, not only this is not a natural assumption but also in the general setting we are considering the stress is put on the case in which $d_L$ is zero on some intervals. In this case $d_L^m=0,$ and the inequality above does not hold.

## Numerical Simulations

We devote this section to illustrate numerically the results presented in Sect. 2. The section will be structured around the two examples previously introduced, one for $R_0>1$ with convergence to a unique limit cycle, and one for $R_0\le 1$ with extinction.

### Example 1: $R_0>1$

In order to illustrate the results presented for $R_0>1$, we simulate system (1) in two different scenarios corresponding to tropical and a temperate climate.

Since the dynamics of mosquito populations depend highly on the climatic conditions, in regions with different climates they can present very different behaviours. We show simulations for two rather different scenarios: The first scenario is based on a tropical area, where temperature is roughly constant throughout the year and there is a dry season and a wet season, causing oscillations in the mosquito population as the wet season is more favorable for reproduction. The second one is based on a more temperate region, with warm summers and cold winters. Mosquito population can thrive during warm summers as long as rain is also present, while low temperatures during winters can make adult population of some species disappear completely. In these cases eggs act as a population reservoir for the next favorable season. With these simulations we want to stress that, as long as the biological parameters are *T*-periodic and satisfy hypothesis (4) to (6), the convergence of solutions to a unique limit cycle, whichever this may be, is guaranteed by Theorem 2.4.

**Fig. 1:**
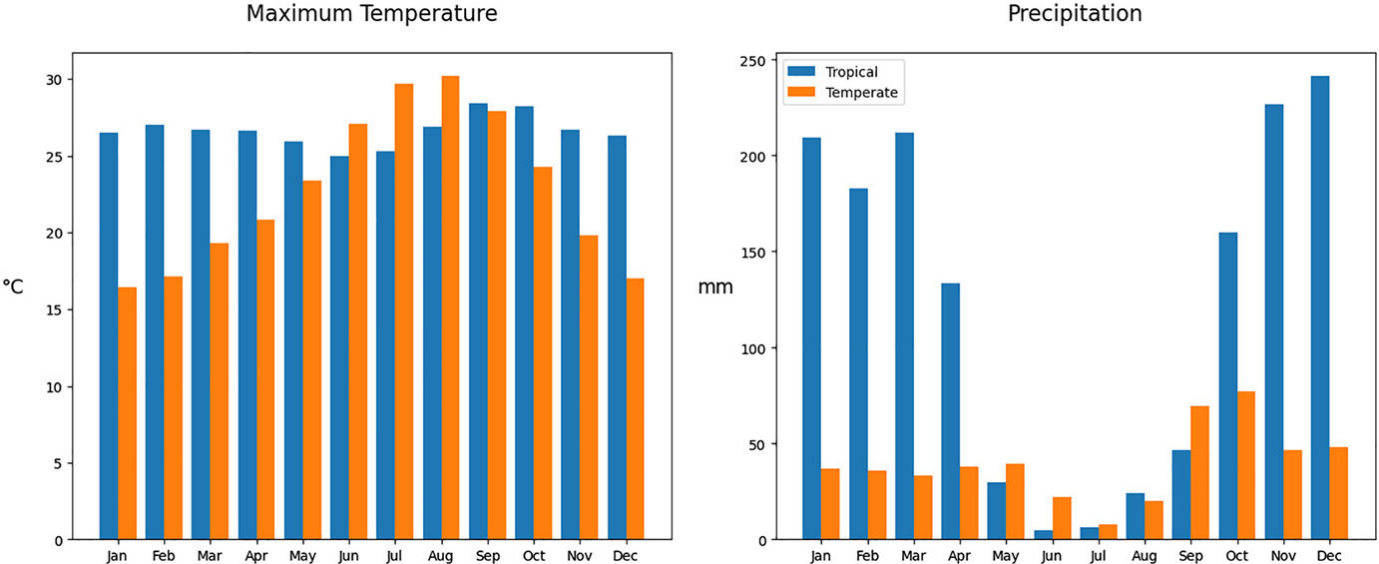
Average maximum temperature and average precipitation per month in a Tropical region (Brasilia) and in a temperate region (Valencia). Reference years 1981–2010 in both cases (Color Figure Online)

**Fig. 2:**
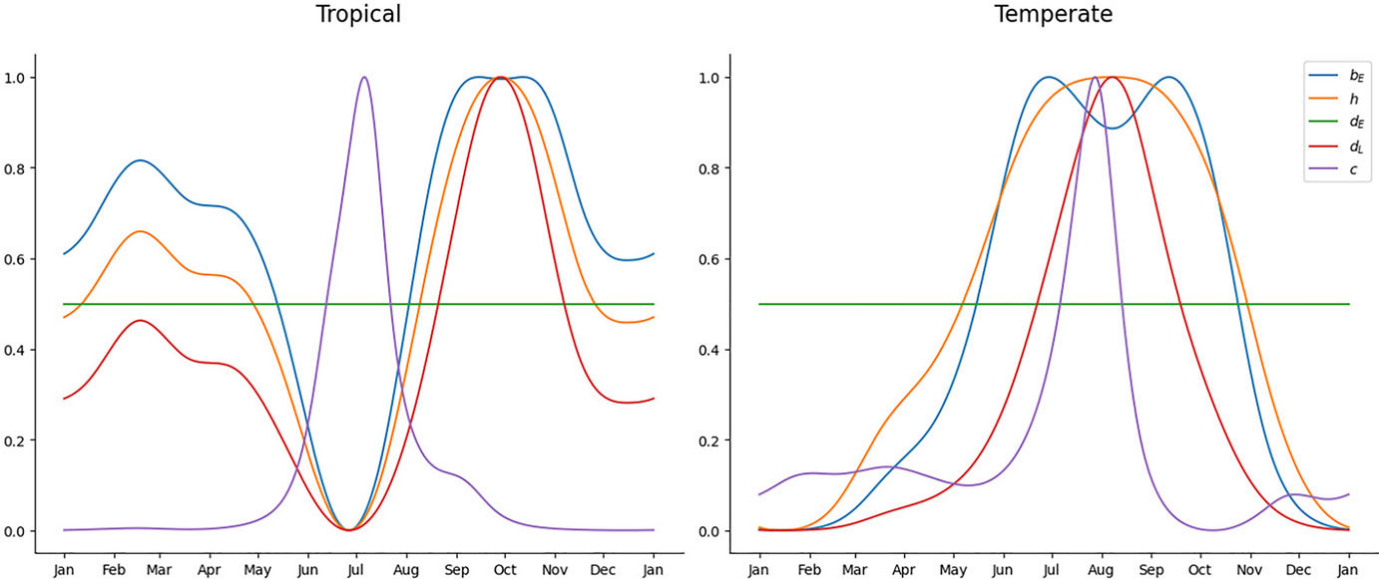
Time dependence of parameters in scenarios 1 (left) and 2 (right). Functions have been normalized to compare purely their periodic behaviours (Color Figure Online)

The periodic nature of the biological parameters comes ultimately from the periodic nature of the climate. We use monthly averaged climatological data from Brasilia, Brazil (Source INMET (Meteorologia 2023a)), and Valencia, Spain (Source AEMET (Meteorología 2023)), see Fig. 1. Interpolating this data, and taking into account the thermal responses of the biological parameters for *Ae. Albopictus* found in Jia et al. (2016), Mordecai et al. (2017), and the modeling of the carrying capacity as a function of the precipitation done in Erguler et al. (2016), we compute the values of the relevant mosquito parameters of system (1) as a function of time (See Fig. 2 and Table 1). There is no consensus on whether maximum, mean or minimum temperatures should be used in order to compute the values of the biological parameters for models like (1). Some works find a similar, and significant, correlation between all of these quantities and mosquito abundance (Tian et al. 2015, Table 1). We will use maximum temperatures for all the parameters in this example. Despite systems (2) and (Jia et al. 2016, 5) not being equal, the relevant temperature-dependent parameters are analogous in both models, except for the oviposition rate. We take the values of these parameters from Tables 3 and 5 in that work. The oviposition rate has been computed as $b_E(t):=\beta _E(t)\frac{\tau _P(t)}{d_A(t)}\frac{\tau _L(t)}{\tau _P(t)+d_P(t)}$, with the thermal response of $\beta _E$ taken from Mordecai et al. (2017). The resulting periodical parameters, of period $T=365$ days, are plotted in Fig. 2.

**Table 1:** Dependences and minimal and maximal values of parameters used in simulations in both scenarios of the Example 1

Parameter	Name	Dependence	Min.- Max. values	Min.- Max. values	Source
			in Scenario 1	in Scenario 2	
$b_E$	Oviposition rate	On temperature	152.33–204.75	5.96–204.75	Mordecai et al. (2017), Jia et al. (2016)
$d_E$	Death rate of the eggs	Independent	0.05–0.05	0.05–0.05	Jia et al. (2016)
$d_L$	Death rate of the larvae	On temperature	0.022–0.029	0.017–0.036	Jia et al. (2016)
*h*	Hatching rate	On temperature	0.41–0.49	0.14–0.51	Jia et al. (2016)
*c*	Larval competition intensity	On precipitation	0.0041–0.29	0.0012–0.014	Erguler et al. (2016)

**Fig. 3:**
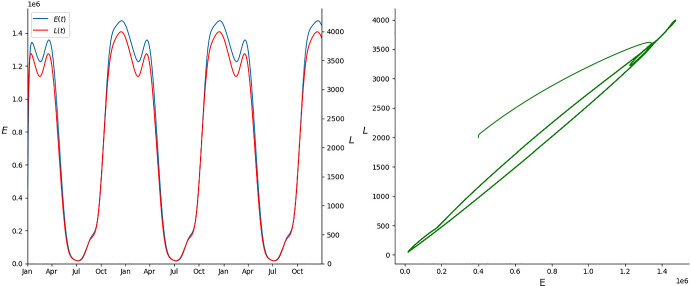
Evolution of the egg and larva population in the first scenario plotted against time (left) and its evolution in the phase space (right) (Color Figure Online)

**Scenario 1.** In this scenario, based on a tropical climate, the maximum temperature remains fairly constant throughout the year, varying from 25 $^{\circ }$C in June to 28.4 $^{\circ }$C in September. Meanwhile, precipitation varies drastically from the dry season (May–September, with a minimum in June of 4.9 mm) to the wet season (October–April, with a maximum in December of 241.5 mm). In this case, the minimum of the oviposition and hatching rates, roughly coincide with the maximum of the larval competition, meanwhile, when the oviposition and hatching rates are at their maximum, the larval competition is close to its minimum. This causes big oscillations between the maximum and the minimum values in the egg and larva population (more than $\pm 85\%$ of the mean value), as we can observe in the left graph of Fig. 3. Hypothesis (4) to (6) are satisfied since all parameters are strictly positive and $\sup _{t\in [0,T]}\frac{d_L(t)}{b_E(t)}\approx 0.00014<0.89\approx \inf _{t\in [0,T]}\frac{h(t)}{d_E(t)+h(t)},$ thus, as proven in Theorem 2.4, we observe the convergence to the unique limit cycle in the right graph of Fig. 3.

**Fig. 4:**
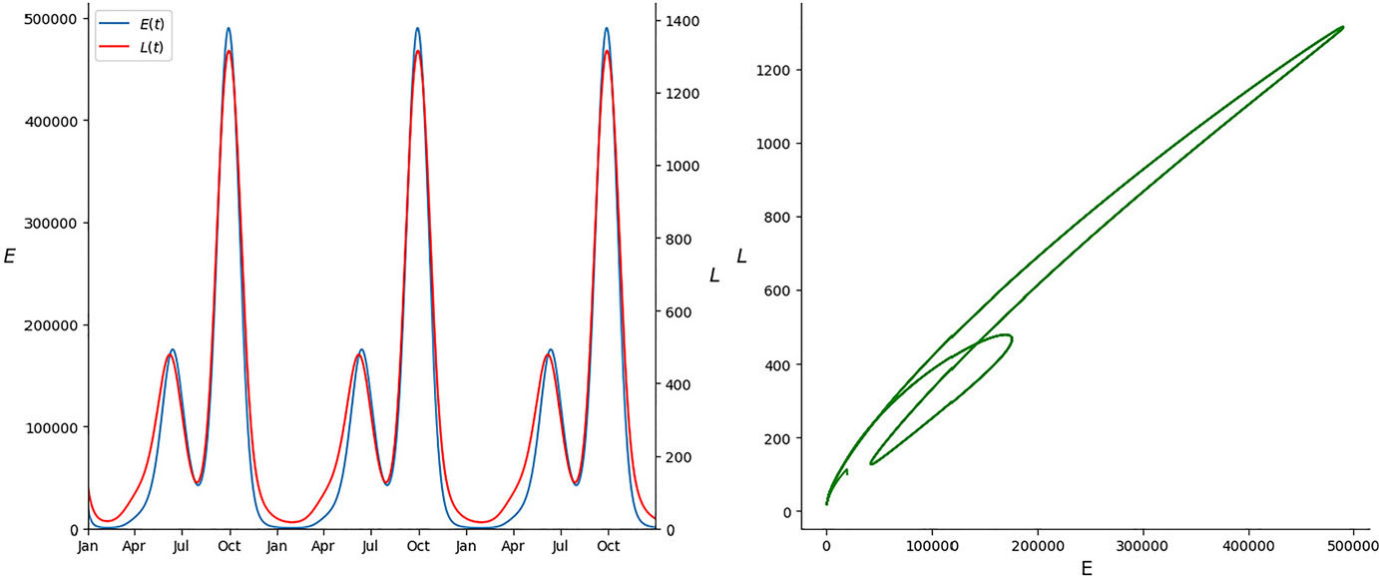
Evolution of the egg and larva population in the second scenario plotted against time (left) and its evolution in the phase space (right) (Color Figure Online)

**Scenario 2.** In this scenario, based on a temperate climate, maximum temperatures oscillate more during the year, ranging from 16.4 $^{\circ }$C in January to 30.2 $^{\circ }$C in August. Rainfall is scarse during summer (June–August, with a minimum of 7.8 mm in July) and abundant in the end of the summer and in autumn (September–December, with a maximum in October of 77 mm). In this setting, warm temperatures and some amount of rainfall at the beginning of the summer cause a first peak in the population. This peak is followed by a valley in August due to a combination of too much heat and too little rainfall. On the other hand, in September and October, heavy rainfall combined with the fact that temperatures are still warm causes a much bigger spike in the population (see the left graph of Fig. 4). Hypothesis (4) to (6) are once again satisfied. In this case $\sup _{t\in [0,T]}\frac{d_L(t)}{b_E(t)}\approx 0.0028<0.74\approx \inf _{t\in [0,T]}\frac{h(t)}{d_E(t)+h(t)}$ and all parameters are strictly positive for all $t\in [0,T]$. The convergence to the unique limit cycle can be visualized in the right graph of Fig. 4.

**Fig. 5:**
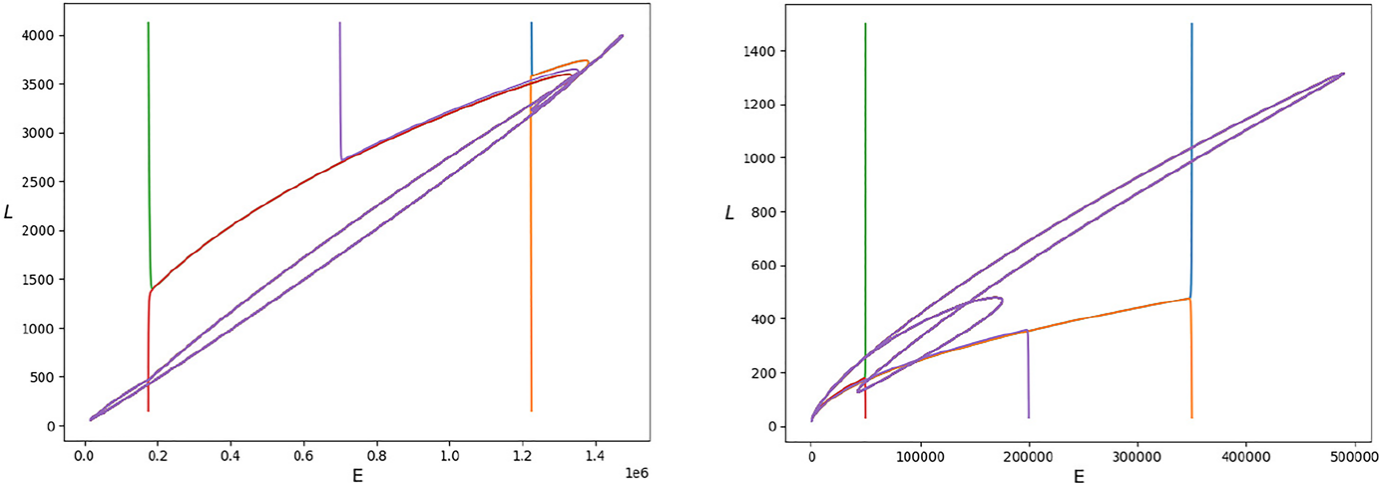
Phase space of solutions to system (1) for different initial conditions in scenarios 1 (left) and 2 (right) (Color Figure Online)

**Convergence to the unique limit cycle.** To illustrate better this central property of the population’s dynamics in case $R_0>1$, in Fig. 5 we plot the phase space of solutions to system (1) in both scenarios for five different initial conditions distributed around the unique limit cycle.

### Example 2: $R_0\le 1$

Here, we illustrate the other behaviour that system (1) can exhibit: extinction. To do this, we follow the steps of the construction carried out in the Example 2 described in Sect. 2.2.

**Fig. 6:**
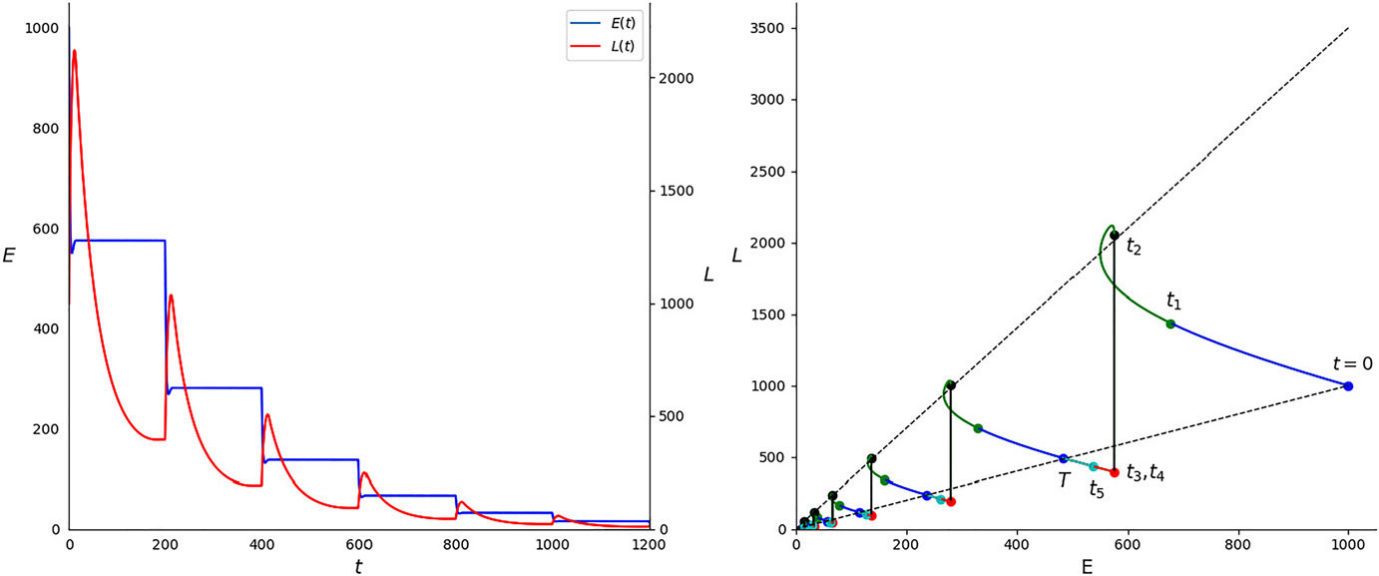
Solution of the linear version of system (1), system (9), in the Example 2 setting plotted against time (left). Phase space of the solution (right) (Color Figure Online)

In Fig. 6 we observe how, as previously shown, egg population remains constant in $(t_2+kT,t_3+kT)$, $k\in \mathbb {N}$, and decreases abruptly in $[kT,t_2+kT]\cup [t_4+kT,(k+1)T]$, giving rise to a spike in the larva population. Next, the larva population decreases exponentially in $(t_2+kT,t_3+kT)$, where $d_L$ is the only positive parameter, in such a way that $L((k+1)T)<L(kT)$. As explained, with this choice of parameters $(E(kT),L(kT))=(\lambda ^k E(0),\lambda ^k L(0))$, with $0<\lambda <1$, therefore ever smaller oscillations occur with each period, and thus the solution tends towards the origin. We plot six full periods. The effects of each step described in Sect. 2.2 can be seen clearly in the plot of the phase space, which corresponds to the right graph in Fig. 6. In this graph, the dotted black lines correspond to $L=\frac{L_0}{E_0}E$ (the lower one) and $L=\frac{\bar{d}_E+\bar{h}}{\bar{b}_E}E$ (the upper one). By comparison, system (1), with the same parameters except for $c>0$, will also go to extinction. The values of the parameters and of the relevant times $t_i$, $i=1,\dots ,5$ and *T* are shown in Table 2.

**Table 2:** Values of the parameters and relevant time stamps in the Example 2 simulations

Parameter	$\bar{b}_E$	$\bar{d}_E$	$\bar{d}_L$	$\bar{h}$	$t_1$	$t_2$	$t_3$	$t_4$	$t_5$	*T*
Value	0.1	0.05	0.02	0.3	2	15	180	199	199.6	200

## Four-Stage Model and Open Problems

It remains open the question of whether the nonautonomous *T*-periodic version of system (2) admits periodic solutions and, in case they exist, what is their stability. In Abdelrazec and Gumel (2017), system

10 $$\begin{aligned} {\left\{ \begin{array}{ll} E'=B(t,A) A-d_E(t) E- h(t) E ,\\ L'=h(t)E-d_L(t) L-c(t) L^2-\tau _L(t) L ,\\ P'= \tau _L(t) L-d_P(t) P-\tau _P(t) P ,\\ A'= \sigma \tau _P(t) P-d_A(t) A, \end{array}\right. } \end{aligned}$$

was considered both in the autonomous and the nonautonomous cases and a very complete analysis was done for the autonomous case. In that work, $\sigma$ represents the proportion of new adult mosquitoes which are females and *A* stands for the total number of adult mosquito females. The oviposition function, *B*(*t*, *A*), was given by one of the following expressions

$$\begin{aligned} {\left\{ \begin{array}{ll} B(t,A)=B_L(t,A)= b(t)\left( 1-\frac{A}{K}\right) ,\,\, A\in [0,K);\\ B(t,A)=B_S(t,A)=\frac{b(t)}{1+\left( \frac{A}{K}\right) ^n},\,\, n>0, \end{array}\right. } \end{aligned}$$

where *K* represents the so called carrying capacity of the environment, in other words, the maximum amount of female mosquitoes that the environment can sustain. In what concerns the nonautonomous model, the existence of a unique coexistence solution was proved only assuming no intraspecific competition for the larvae *c*(*t*).

In this work, we will consider just the oviposition function $B_S$ with $n=1$, namely

11 $$\begin{aligned} B_S(t,A)=\frac{b(t)}{1+\frac{A}{K}}, \end{aligned}$$

with *b*(*t*) continuous, $T-$ periodic and strictly positive on [0, *T*].

For the remaining coefficients in (10), we assume that they are continuous, *T*-periodic, nonnegative and, with the possible exception of *c*,  have positive integral in [0, *T*]. In other words, now one can have $c(t)=0$ for all $t\in [0,T]$, contrary to what was considered for system (1). This assumption was used in Sect. 1 to prove the dissipativity of the system (1) and the strict subhomogeneity of the corresponding vector field. Here the dissipativity can be obtained under other assumptions on the remaining coefficients, and the subhomogeneity is given by $B_S.$ As a consequence, we can include in our setting the case in which there is no intraspecific competition for the larvae. Moreover, we assume that the function

12 $$\begin{aligned} \mathcal{{M}}(t):=\min \{d_E(t), d_L(t), d_P(t), d_A(t)\} \end{aligned}$$

has positive average on [0, *T*].

Finally, we denote by $F=(F_1,F_2,F_3, F_4)$ the vector field associated with system (10) and denote by *DF*(*t*, *E*, *L*, *P*, *A*) its Jacobian matrix with respect to the variables (*E*, *L*, *P*, *A*).

Below, using again Zhao (2017, Theorem 3.1.2), we will obtain a result analogous to Theorem (2.4) for system (10) with the oviposition rate (11) but allowing nonzero intraspecific competition, so generalizing the corresponding results in Abdelrazec and Gumel (2017).

As far as we know, the case in which *c* is not zero remains open for $B_L$ and $B_S$ with $n>1.$

First of all, we state a lemma, analogous to Lemma 2.1, whose proof is also analogous and follows immediately from the property:

if $x=(x_1,x_2,x_3,x_4):=(E,L,P,A)\ge 0,\,$ then $x_i=0 \Rightarrow F_i(t,x)\ge 0,\,\,\,i=1,2,3,4,\,$ for each $t\ge 0$.

### Lemma 4.1

Let (*E*(*t*), *L*(*t*), *P*(*t*), *A*(*t*)) be a solution of system (10) with initial condition $(E(0),L(0),P(0), A(0))\ge 0$. Let $I:=[0,\omega )$ be its maximal right domain of existence. Then, $(E(t),L(t),P(t),A(t)) \ge 0$ for all $t\in I.$

In Abdelrazec and Gumel (2017, Theorem 2.1) it is stated that solutions of (10) with nonnegative initial conditions are bounded. We give a sketch of the proof of this result for completeness.

### Lemma 4.2

Solutions of (10) are defined for each $t\ge 0$. Moreover, there exists a $\tilde{\mathcal{K}}>0$ such that $E(t)+L(t)+P(t)+A(t)\le \tilde{\mathcal{K}}$ for each *t* sufficiently large.

### Proof

The proof is analogous to the one of Lemma 2.2. We have

$$\begin{aligned} \frac{d}{dt}(E+L+P+A)&\le B(t,A)A-\mathcal{{M}}(t)(E+L+P+A) \\&\le K b^M-\mathcal{{M}}(t)(E+L+P+A) \end{aligned}$$

where, as previously, $b^{M}$ is the maximum of *b*(*t*). Then, taking into account (12), our claim is obtained as in Lemma 2.2. $\square$

Now we are able to prove that solutions with nonnegative initial condition will be positive at 3*T*.

### Corollary 4.3

Let (*E*(*t*), *L*(*t*), *P*(*t*), *A*(*t*)) be a solution of system (10) with initial condition $(E(0),L(0),P(0), A(0))\ge 0$. Then, if the the initial condition is not the origin, $(E(3T),L(3T), P(3T), A(3T))>0$.

### Proof

The proof follows the steps of the proof of Lemma 2.1 and hence we only give a sketch. If one of the components of the initial condition is positive for some $\bar{t}$, that component remains positive for all $t\ge \bar{t}$. Let us assume now, as the extreme case, that only one of the components of the initial condition is positive, let us say $E(0)>0$. Then, considering the second equation of (10), since *h* has positive average on [0, *T*] we see that there exists exists $t_1\in [0,T)$ such that $L(t)>0$ for each $t>t_1$. We turn now to the third equation of (10) and argue in a similar manner: since $\tau _L(t)$ has positive average on [0, *T*] we conclude that there exists $t_2\in [T,2T)$ such that *P*(*t*) for $t>t_2.$ Finally, since $\tau _P$ has positive average on [0, *T*],  we get that there exists $t_3\in [2T,3T)$ such that such that $A(t)>0$ for any $t>t_3.$ We conclude that the solution will be positive at time 3*T*.

The case in which the only nonzero component of the initial condition corresponds to one of the other stages is proved analogously. Actually, if the unique nonzero component is one of the others the corresponding solutions will be positive at time 2*T*. In any case, all the solutions corresponding to one positive initial component will be positive at time 3*T*. $\square$

In what follows we will need a positive operator and hence we will use the 3*T*- Poincaré map, $\mathcal{{P}}_{3T}$, instead of the *T*-Poincaré map. Of course, $\mathcal{{P}}_{3T}=\Pi _T^3,$ where $\Pi _T$ denotes the *T*-Poincaré map of system (10).

We define analogously (see Sect. 2 and Abdelrazec and Gumel 2017) the vectorial reproduction number $R_0^{3T}$ and recall that $R_0^{3T}=(R_0)^3$. We can finally obtain the following result which generalizes (Abdelrazec and Gumel 2017, Theorems 4.2, 4.3).

### Theorem 4.4

We have that

- If $R_0\le 1$ then for each nonnegative initial condition, the corresponding solution of (10) $(E(t),L(t), P(t), A(t))\rightarrow (0,0,0,0)$.
- If $R_0>1$ then there exists a unique positive $T-$periodic solution $(E^*(t),L^*(t),P^*(t),A^*(t))$ of (10) which is globally asymptotically stable in $\textbf{R}_+^4{\setminus }\{(0,0,0,0)\}$.

### Proof

The result follows from Zhao (2017, Theorem 3.1.2). We have that property (i) holds together with the following ones:

(ii) $$\frac{\partial F_i}{\partial x_j}\ge 0$$ for $$i \ne j.$$
(iii) *F* is strictly subhomogeneous.

Property (ii) is immediate. Property (iii) holds since $\alpha B_S(t,A)>B_S(t,\alpha A)$ for any $\alpha \in (0,1), \,t\ge 0,\,A>0.$ Moreover, by Corollary 4.3 we know that $\mathcal{{P}}_{3T}$ is positive. To see that $\mathcal{{P}}_{3T}$ is also strongly positive we proceed as in the proof of Theorem 2.4, arguing analogously as in the proof of Corollary 4.3 to show that its Jacobian matrix $D\mathcal{{P}}_T$ has all positive entries. The difference with the Lemma is that, when $E(0)=0,$ to get that *E*(*t*) becomes positive we must use the fact that the entry

$$\begin{aligned} \frac{\partial (B_S(t,A)A)}{\partial A}=\frac{b(t)}{\left( 1+\frac{A}{K}\right) ^2} \end{aligned}$$

of *DF*(*t*, *E*(*t*), *L*(*t*), *P*(*t*), *A*(*t*)),  is positive whenever $A(t)>0$ (a property that does not hold for $B_S$ for $n>1$). Again, we do not need the irreducibility condition on the vector field. We conclude that we can apply Wang and Zhao (2008, Theorem 2.2) and obtain the result for $R_0^3$ instead of $R_0$ (but $R_0<1$ if, and only if, $R_0^3<1$) and the existence of a unique 3*T*-periodic orbit which attracts all the solutions with nonzero initial condition. Now let $Z_0$ be the initial condition of this periodic orbit. Then $\mathcal{{P}}_{3T}(Z_0)=Z_0$ so that $\Pi _T^3(\Pi _T(Z_0))=\Pi _T(Z_0)$ which, since the fixed point $Z_0$ is a globally asymptotically stable fixed point of $\Pi _T^3$, implies that $\Pi _T(Z_0)=Z_0$. Now we conclude that the 3*T*-periodic orbit is *T*- periodic and the result follows. $\square$

## Discussion

In this paper we considered the two-stage model with periodic coefficients (1) and we were able to give a complete description of its dynamics in terms of $R_0.$

An autonomous version of this system in which the hatching rate depends on the larval density was studied in Strugarek et al. (2019). The autonomous system in Strugarek et al. (2019) was obtained from a four stage one using a procedure that, in our case, should be applied to the seasonal version of system (2). In this work, as a first step, we addressed just the *T*-periodic two-stage system. We considered a general mathematical framework, which includes biologically meaningful scenarios, in which some time periodic rates can be zero on some time intervals. We proved that either system (1) admits a unique limit cycle, and hence a unique oscillatory regime, if $R_0>1$, or there is extinction if $R_0\le 1$. We showed that the oscillatory regime may occur in both tropical or temperate regions, since in both cases, for a realistic set of parameters, the periodic coefficients satisfy assumptions (4) to (6) of Theorem 2.4. The numerical simulations show clear qualitative differences in the nature of the oscillations in the two scenarios. Our analysis also gives some insight into the possible mechanisms which lead to extinction, showing that extinction may occur if the death rate $d_L$ is positive when the hatching rate *h* is zero (in which case either (4) or (6) do not hold).

In what concerns system (2) with seasonality, we were not able to prove the existence of a bounded set which attracts all the orbits when $R_0\ge 1$ and hence we are neither able to prove the existence and uniqueness of a globally asymptotically stable *T*-periodic positive orbit nor to study the case $R_0=1.$ In the case $R_0>1$, numerical simulations suggest that the globally asymptotically stable *T*-periodic positive orbit exists, but from a theoretical point of view, this remains an open problem. As for the case $R_0<1,$ we observe that, the system being monotone, the global asymptotic stability of the origin may be proved directly, by comparison with the linear system obtained by neglecting the $-c(t)L^2$ term.

In the case of system (10), we were able to prove the dynamics in terms of $R_0$ in the case with intraspecific competition among the larvae, generalizing the results in Abdelrazec and Gumel (2017) in the case of the oviposition function $B_S$ with $n=1$.

In Liu et al. (2017) a four stage model with delays for the ticks life cycle was considered but when intraspecific competition between adults is present. Analogous results were obtained, but the global stability of the periodic orbit was obtained only when there was no intraspecific competition in the first stages. One year after, in Wang and Zou (2018), an analogous model was addressed but not considering intraspecific competition, and the solutions can be unbounded. In this case results of persistence were obtained but not of stability of periodic orbits. In Lv et al. (2021), when intraspecific competition in adults is present, an analogous model with diffusion and delays was analysed and also in this case the existence of an attracting periodic orbit was proved but when no intraspecific competition in larvae is considered. Hence the case when this competition is considered remains an open problem.
